# Genome-Wide Classification and Evolutionary and Expression Analyses of Citrus *MYB* Transcription Factor Families in Sweet Orange

**DOI:** 10.1371/journal.pone.0112375

**Published:** 2014-11-06

**Authors:** Xiao-Jin Hou, Si-Bei Li, Sheng-Rui Liu, Chun-Gen Hu, Jin-Zhi Zhang

**Affiliations:** Key Laboratory of Horticultural Plant Biology (Ministry of Education), College of Horticulture and Forestry Science, Huazhong Agricultural University, Wuhan, China; NARO Institute of Fruit Tree Science, Japan

## Abstract

*MYB* family genes are widely distributed in plants and comprise one of the largest transcription factors involved in various developmental processes and defense responses of plants. To date, few *MYB* genes and little expression profiling have been reported for citrus. Here, we describe and classify 177 members of the sweet orange *MYB* gene (*CsMYB*) family in terms of their genomic gene structures and similarity to their putative *Arabidopsis* orthologs. According to these analyses, these *CsMYBs* were categorized into four groups (4R-MYB, 3R-MYB, 2R-MYB and 1R-MYB). Gene structure analysis revealed that 1R-MYB genes possess relatively more introns as compared with 2R-MYB genes. Investigation of their chromosomal localizations revealed that these *CsMYBs* are distributed across nine chromosomes. Sweet orange includes a relatively small number of *MYB* genes compared with the 198 members in *Arabidopsis*, presumably due to a paralog reduction related to repetitive sequence insertion into promoter and non-coding transcribed region of the genes. Comparative studies of *CsMYBs* and *Arabidopsis* showed that *CsMYBs* had fewer gene duplication events. Expression analysis revealed that the *MYB* gene family has a wide expression profile in sweet orange development and plays important roles in development and stress responses. In addition, 337 new putative microsatellites with flanking sequences sufficient for primer design were also identified from the 177 *CsMYBs*. These results provide a useful reference for the selection of candidate *MYB* genes for cloning and further functional analysis forcitrus.

## Introduction

MYB proteins constitute one of the largest families of plant-specific transcription factors, and the family is present in a wide range of land plants. They are characterized by a structurally conserved DNA-binding domain consisting of single or multiple imperfect repeats located near the N-terminus, which can function synergistically or individually in DNA binding and protein–protein interactions, respectively [1]. Each repeat is approximately 50 to 53 amino acids and encodes three α-helices, with the second and third helices forming a helix–turn–helix (HTH) structure that intercalates in the major groove of DNA when binding to it [2]. Different categories of MYB proteins can be identified depending on the number of imperfect repeats of the MYB domain they contain: 4R-MYB has four repeats; 3R-MYB (R1R2R3-MYB) has three consecutive repeats; 2R-MYB (R2R3-MYB) has two repeats; and the 1R-MYB (MYB-related type) usually, but not always, has a single repeat [3], [4]. All four classes are found in plants, thus representing the taxon with the highest diversity of MYB proteins. In animals, 3R-MYB proteins are predominant, but the 2R-MYB proteins are more prevalent in plants [3], [5]. The plant-type 2R-MYB genes probably evolved from the 3R-MYB genes progenitor through loss of R1 repeat or from an MYB-related gene through duplication of R1 repeat [6], [7].

Since the first gene (*COLORED1*) encoding a *MYB* transcription factor was identified in maize [8], research concerning different aspects of the *MYB* gene family, including gene number, sequence characterization, evolution, and potential functions, has been widely conducted in plants [1], [9]–[11]. So far, large numbers of *MYB* genes have been identified in different plants, with 198 members in *Arabidopsis*, 183 members in rice, 279 members in grapevine, 197 members in poplar and 180 members in Brachypodium [11]–[14]. Although *MYB* genes have also been identified in woody species such as poplar and grapevine, *MYB* genes have been much less studied in woody species than in many herbaceous species. Numerous MYB proteins have been characterized by genetic approaches and have been found to be involved in many significant physiological and biochemical processes, including developmental control and determination of cell fate and identity, plant responses to environmental factors and hormones, signal transduction in model plant growth processes, abiotic stress and pathogen defense [4], [15]–[17]. Other functions of *MYB* include regulation of secondary metabolism, the cell-cycle regulation, xylogenesis biosynthesis during wood formation, and skin, flesh, and foliage anthocyanic color [18]–[22]. Furthermore, recent studies have shown that the *MYB* genes are post-transcriptionally regulated by microRNAs; for example, *AtMYB33*, *AtMYB35*, *AtMYB65*, and *AtMYB101* genes involved in anther or pollen development are targeted by the miR159 family in *Arabidopsis* [23], [24].

Citrus is the fruit crop with the highest production worldwide; therefore, the citrus industry has great economic importance. Some citrus traits might be the consequence of different *MYB* gene networks based on previous studies, compared with its herbaceous counterparts *Arabidopsis* and rice. In previous studies, some members of the *MYB* gene were identified based on cDNA library and specific probes designed from the conserved MYB domain in citrus [19], [25], but a genome-wide analysis of the *MYB* gene family has not yet been undertaken for citrus. Recently, the sweet orange genome has been sequenced [26]–[28], and the completion of genome provided an opportunity for us to analyze the *MYB* gene family in citrus. Knowledge of the complete genome sequence also provides a valuable resource for comparative analyses, which can be used to further understand the evolutionary history of the *MYB* family genes. Therefore, we performed a genome-wide survey of the *MYB* gene family in sweet orange. A total of 177 open reading frames (ORFs) encoding *MYB* genes were identified, most of which remain to be functionally characterized. A phylogenetic tree combining sweet orange and *Arabidopsis* MYB proteins was constructed to examine their evolutionary relationships and the putative functions of citrus MYB proteins based on *Arabidopsis* MYB proteins with known functions. Tissue-specific gene expression analysis was conducted and stress expression profiles were generated to find potential genes that participate in the stress signal transduction pathway in citrus. This extended analysis was the first comprehensive study of the *MYB* gene family in sweet orange and provided valuable information for further exploration of the functions of this significant gene family in citrus.

## Results

### Identification and classification of *CsMYBs*

To identify *MYB* encoding genes in sweet orange, two different HMM profiles were performed. Several deduced amino acid sequences (>300 candidates) containing MYB or MYB-like repeats were obtained. Only hits with E values <1.0 were considered members of this gene family. Subsequently, the redundant sequences of candidate sweet orange MYB genes (*CsMYBs*) were discarded from our data set according to their chromosome locations. In addition, the remaining *CsMYBs* possessing incomplete ORFs were also excluded from further analysis. Finally, a total of 177 non-redundant *CsMYBs* were identified (Table S1). We provisionally named them *CsMYB1* to *CsMYB177*, based on the order of the corresponding chromosome locations identified from the sweet orange genome browser in each category (http://citrus.hzau.edu.cn/cgi-bin/gb2/gbrowse/orange/). We classified *CsMYBs* in four distinct groups 1R-MYB, 2R-MYB, 3R-MYB, and 4R-MYB based on the presence of one, two, three and four MYB repeats, respectively (Table S1). Our analysis revealed that 1R-MYB type consisted of the highest number of *MYB* genes, with 50.85% (90 genes) of the total *MYB* genes. The 2R-MYB type represented 48.02% (85 genes) of the total *CsMYBs*, and thus constituted the second largest group of *MYB* genes. In contrast, only a few 3R-MYB type genes and 4R-MYB type genes were identified in sweet orange, with one gene (*CsMYB86*) and one gene (*CsMYB87*) genes, respectively (Table S1).

### Gene structure of *CsMYBs*

**Figure 1:**
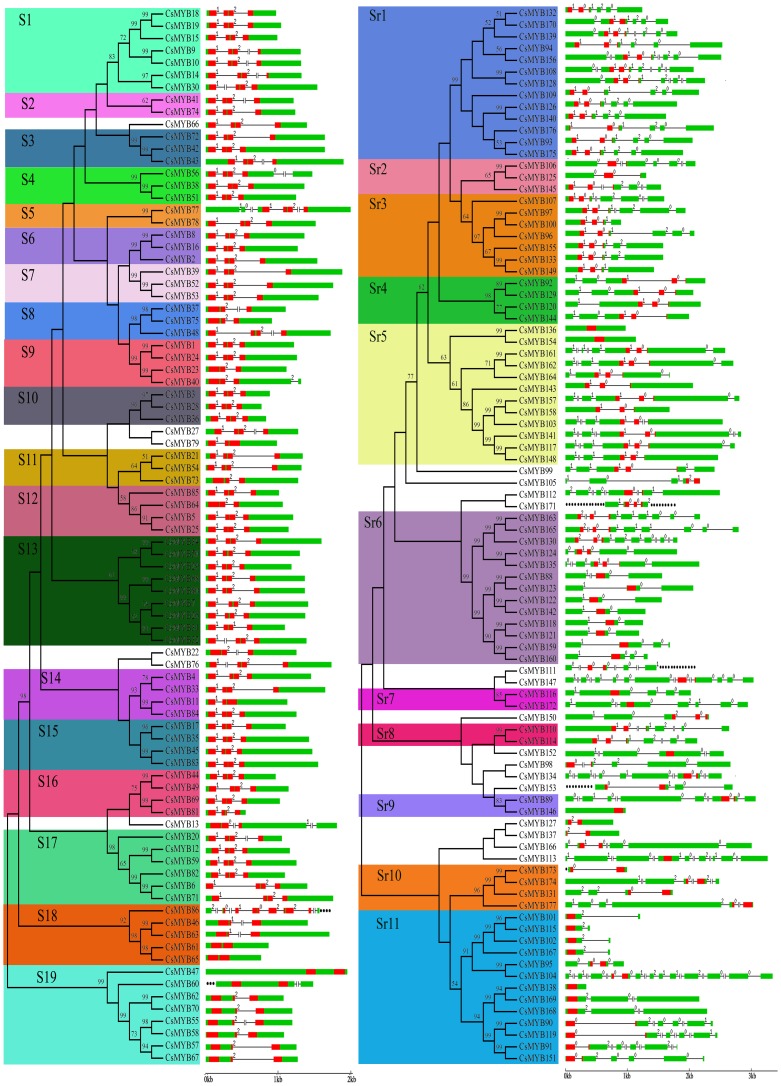
Phylogenetic relationship and intron pattern in CsMYB proteins. The neighbor-joining (NJ) tree on the left included 177 CsMYB proteins from sweet orange. For 86 R2R3-MYB and 3R-MYB proteins, the tree showed the 18 phylogenetic subgroups (S1–S18) marked with colored backgrounds to facilitate subfamily identification with high predictive value. Six proteins did not fit well into clusters. For 91 1R-MYB and 4R-MYB proteins, the tree shows the 11 phylogenetic subgroups (Sr1–Sr11) marked with colored backgrounds, to facilitate subfamily identification with a high predictive value. Fifteen proteins did not fit well into clusters. The numbers beside the branches represent bootstrap values (50%) based on 1,000 replications. The colorful marker in the tree indicates the corresponding intron distribution patterns. The gene structure was presented by green exon(s), red MYB domain(s), and spaces between the colorful boxes corresponding to introns. The sizes of exons and introns can be estimated using the horizontal lines; the numbers indicate the phases of corresponding introns.

To understand the structural component of *CsMYBs*, their exon and intron organizations were obtained by comparing the cDNA sequences with the corresponding genomic DNA sequences (Fig. 1). The number of introns per ORF varied, with a maximum of 26 (*CsMYB171*) and a minimum of 0 (*CsMYB136*) introns (Table S1). The *CsMYB115* contained the shortest introns (89 bp) and the *CsMYB161* contained the longest intron (5,889 bp). The closely related genes were generally more similar in gene structures, differing only in intron and exon lengths (Fig. 1). But some close gene pairs were distinct in intron-exon arrangements. For example, *CsMYB104* contained 13 exons, whereas its close paralogs *CsMYB95* had only four even though their phylogenetic relationship was supported by a high bootstrap value. In addition, the intron positions on MYB domains were investigated. We noticed that a large number (73; 85.88%) of-2R MYB proteins have a conserved splicing pattern with three exons and two introns (Fig. 1). However, some 2R-MYB genes lack one intron, in either R2 or R3 repeat (Fig. 1). It has been proposed that the duplication of 2R in an early form of two repeat MYB proteins gave rise to the 3R-MYB domains. Hence, we also further investigated the exon-intron structure of 3R-MYB proteins. We observed that 3R-MYB proteins (*CsMYB86*) contained a conserved pattern of three exons-two introns in R1 and R2 and one conserved intron in R3 repeat (Fig. 1). Phylogenetic analysis of *CsMYBs* showed that the gene of the same subgroups generally contained the same intron pattern, with the position(s) of the intron(s) being fully conserved (Fig. 1).

### Analysis of protein properties and conserved motif

To further understand the molecular characteristics of CsMYB proteins, their physiochemical properties were analyzed. We observed that the *CsMYBs* encode proteins ranging from 95 to 1,763 amino acids in length with an average of approximately 386.5 amino acids, and the average molecular weight was approximately 43 kDa (Table S2). The mean pI values for 1R-MYB, 2R-MYB, 3R-MYB, and 4R-MYB protein families were 7.23, 6.81, 5.18 and 8.37, respectively (Table S2). The average molecular weight of 1R-MYB, 2R-MYB, 3R-MYB, and 4R-MYB proteins were 47.26, 37.42, 114.3 and 109.04 kDa. We also observed that all CsMYB proteins had a negative grand average hydropathy (GRAVY) score, suggesting that CsMYB proteins are soluble proteins, which is a character that is necessary for transcription factors. Minimum and maximum GRAVY scores were recorded as −1.33 (orange1.1t02012.1) and −0.082 (Cs2g01900.1), respectively (Table S1). In addition, the subcellular localization of CsMYB proteins was predicted using localization predictor software. The results revealed that 94.35% of CsMYB proteins were found to be nuclear localized and confirmed by the presence of nuclear-localization signal (NLS). The remaining 10 members of CsMYB proteins were predicted to be localized in mitochondria, plasma membrane and chloroplast (Table S1).

**Figure 2:**
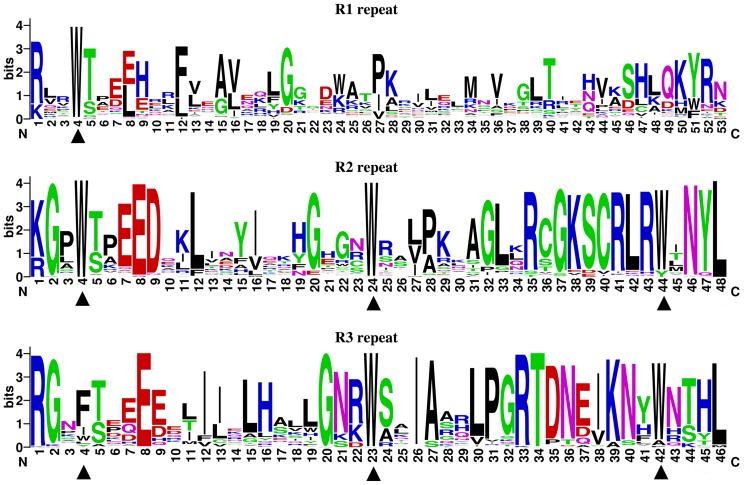
Three Repeats of MYB in the sweet orange genome. The R1, R2 and R3 MYB repeats were highly conserved across all CsMYB proteins in the sweet orange genome. The sequence logos of the R1, R2 and R3 MYB repeats were based on full-length alignments of all CsMYB proteins. The overall height of each stack indicates the conservation of the sequence at that position, whereas the height of letters within each stack represents the relative frequency of the corresponding amino acid. ▴ indicates the conserved tryptophan residues (Trp) in the MYB domain.

Sequence logos of the MYB domain were produced to examine the level of conservation of R1, R2, and R3 repeats in the CsMYB proteins within each residue position. The results reveal that one, two, and one conserved amino acid residues were identical among all the members detected in the R1, R2, and R3 MYB repeat regions, respectively (Fig. 2). The residues had different levels of conservation in the other positions, and approximately half of them had>50% appearance at the respective position (Fig. 2). For the 177 CsMYB proteins, all of the R1 repeat sequences contained one tryptophan residue and almost all of R2 and R3 repeat sequences contained three tryptophan residues. However, the first tryptophan residue was replaced by phenylalanine, and the second and third tryptophan residues were conserved in almost all the members in the R3 repeats (Fig. 2).

To further reveal the diversification of CsMYB proteins, putative motifs were also predicted by the MEME program, and 15 distinct motifs were identified in 90 1R-MYB proteins and in 1 4R-MYB protein and designated as motifs 1 through 15 (Fig. S1). Meanwhile, 15 distinct motifs were also identified in 85 2R-MYB proteins and in 1 3R-MYB protein and designated as motifs 1 through 15(Fig. S2). The length of motifs varies from 8 to 107 amino acids and the number of motifs in each CsMYB protein varies between 0 and 5 (Fig. S1, Fig. S2). In 1R-MYB and 4R-MYB proteins, motif 2 was the most common motif, which was found in 65 CsMYB proteins (Table S3). The next common motifs were motifs 1 and 9, present in 43 and 30 CsMYB proteins, respectively (Table S3). In 2R-MYB and 3R-MYB proteins, motif 3 was the most common motif, which was found in 86 2R-MYB and 3R-MYB proteins (Table S4). The next common motifs were motif 2 and motif 1 present in 77 and 72 2R-MYB proteins, respectively (Table S4). We revealed that most members of the same subgroup shared one or more identical motifs outside the MYB DBD domain. These results also may indicate that the highly conserved protein motifs were protein domain combinations that were often lineage-specific. Although most of these conserved motifs remain to be functional elucidated, it is likely that some play important roles in the transcriptional regulation of target genes and may promote further functionally diversification in specific lineages. Taken together, the results suggest that these motifs were evolutionarily conserved and functionally important.

### Comparative phylogenetic and functional analyses of *CsMYBs*

**Figure 3:**
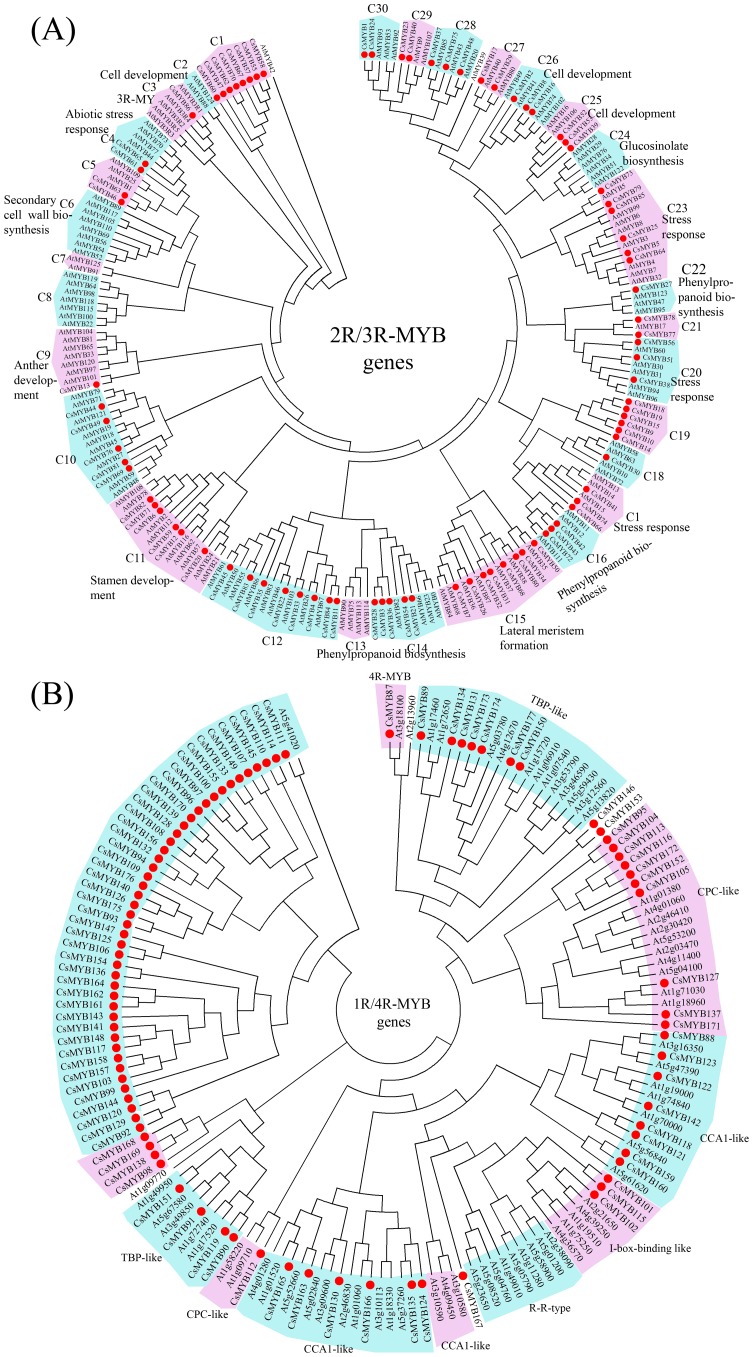
Phylogenetic relationships. (A) Phylogenetic relationships of sweet orange and *Arabidopsis* 2R-MYB and 3R-MYB proteins. The complete amino acid sequences of the 86 sweet orange and 131 *Arabidopsis* 2R-MYB and 3R-MYB proteins were aligned by ClustalW, and the neighbor–joining tree was constructed using MEGA 4.1 with 1000 bootstrap replicates. The functions of some clades were annotated. (B) Phylogenetic relationships of sweet orange and *Arabidopsis* 1R-MYB and 4R-MYB proteins. The complete amino acid sequences of the 91 sweet orange and 67 *Arabidopsis* 1R and 4R-MYB proteins were aligned by ClustalW, and the neighbor–joining tree was constructed using MEGA 4.1 with 1,000 bootstrap replicates. The functions of some clades were annotated. Red circles indicate *MYB* gene from sweet orange.

The functions of several *Arabidopsis* MYB proteins have been well-characterized experimentally [3], [10]. Therefore, a phylogenetic tree combining sweet orange and *Arabidopsis* MYB proteins not only would help to understand the phylogenetic relationships of CsMYB proteins but also would allow speculation on the putative functions of the CsMYB proteins. Previous studies indicated that 1R-MYB proteins had undergone differentiation and evolution to a larger extent than 2R-MYB, 3R-MYB and 4R-MYB proteins in plants [14]. Therefore, the phylogenetic trees for 1R-MYB and 4R-MYB protein subfamilies (Fig. 3A) and the 2R-MYB and 3R-MYB subfamilies (Fig. 3B) were constructed. The resulting trees generated 30 clades for sweet orange 2R-MYB and 3R-MYB subfamilies (named C1–C30), and 12 clades for the sweet orange 1R-MYB and 4R-MYB subfamily, respectively (Fig. 3). A total of 174 of the 177 CsMYB proteins were clustered into 34 clades. The other three CsMYB proteins (CsMYB146, CsMYB153 and CsMYB167) did not fit into any clade (Fig. 3). Remarkably, 30 clades included different numbers of MYB proteins from the two species, whereas another four clades only contained CsMYB proteins (Fig. 3). In some cases, it was easy to distinguish the putative orthologous genes of *CsMYBs* in *Arabidopsis* because they were grouped in pairs within a clade. Most CsMYB proteins were composed of a set of conserved motifs in the C-terminal and the protein architectures were remarkably conserved within specific subgroups. The schemes of protein motifs of individual members of the *MYB* gene family indicated structural similarities within subgroups, further supporting the subgroup definition in phylogenetic analysis.

To investigate biological processes possibly regulated by 177 *CsMYBs*, GO annotation of these genes was performed by Blast2GO [29]. Figure S3 wholly summarizes the categorization of these *CsMYBs* according to the biological process, cellular component and molecular function. Based on biological processes, 177 *CsMYBs* were classified into 25 categories (Fig. S3A); the most over-represented GO term was regulation of DNA-dependent transcription (29 genes). A large number of genes with known functions were classified in response to hormonal stimulus (salicylic acid: 10 genes; jasmonic acid: 9 genes; ethylene: 7 genes; abscisic acid: 7 genes; gibberellin: 5 genes; auxin: 4 genes; and cytokinin: 3 genes), growth and development (stamen: 4 genes; root: 4 genes; cell: 3 genes; trichome differentiation: 3 genes; anatomical structure morphogenesis: 3 genes; and cell wall organization or biogenesis: 3 genes), and signaling pathways (intracellular signal transduction: 3 genes; phosphorelay signal transduction system: 3 genes and hormone-mediated signaling pathway: 3 genes). These results suggest that *CsMYBs* were involved in a broad range of physiological functions. It will be interesting to identify the functions of these *CsMYBs* in citrus. Categories based on molecular function revealed that these *CsMYBs* were classified into seven groups in the species under observation (Fig. S3B); four of the groups were DNA binding (76 genes), chromatin binding (72 genes), sequence-specific DNA binding transcription factor activity (22 genes), and zinc ion binding (5 genes). Based on the cellular component, these *CsMYBs* were related to the nucleus in sweet orange, consistent with the prediction using localization predictor software (Fig. S3C).

### Distribution of *MYB* domain-containing proteins in plant kingdom

**Figure 4:**
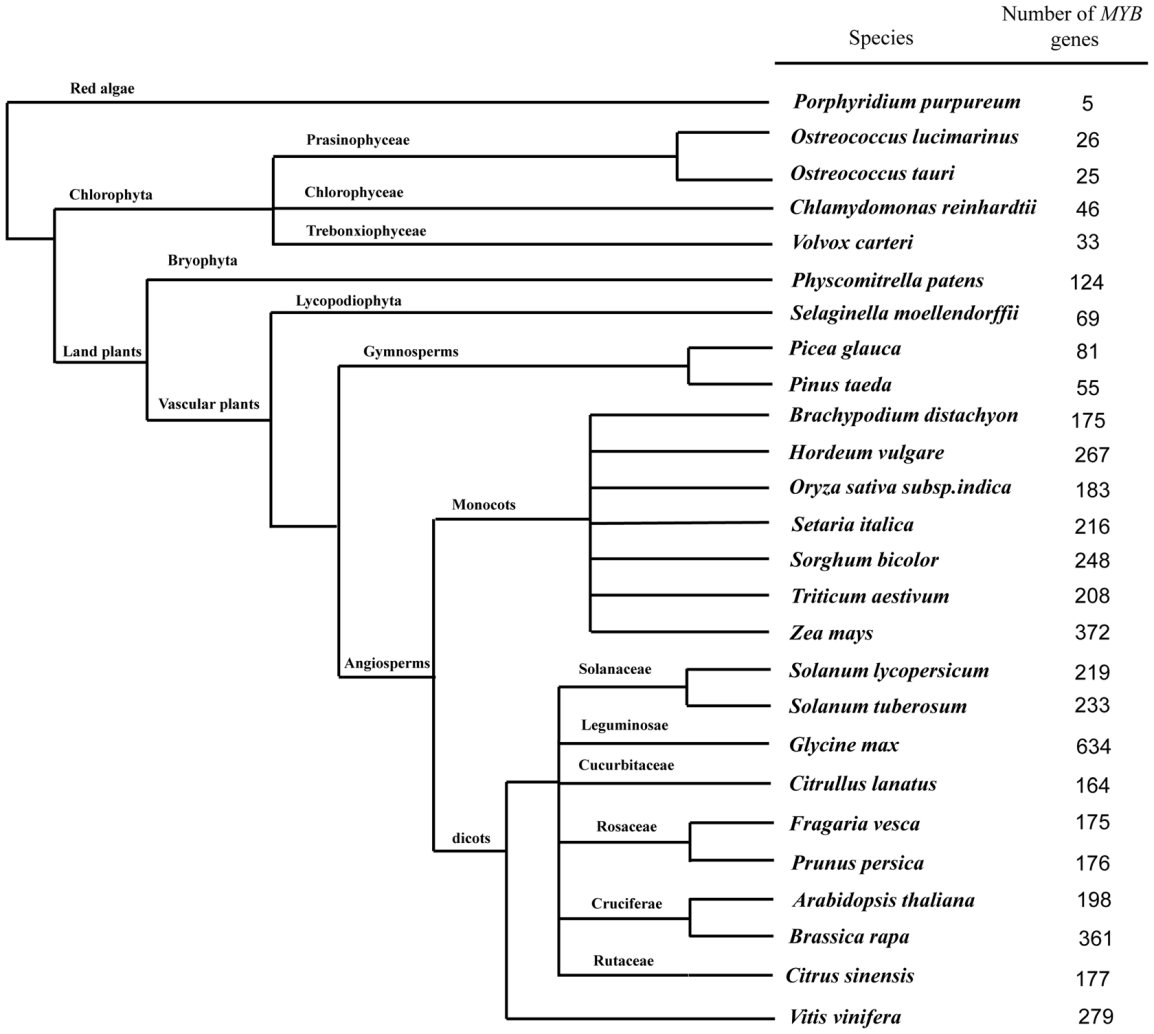
Phylogenetic relationships between all species investigated in this study. The total number of MYB-related proteins found in each genome is indicated on the right. For the number of *MYB* genes in this figure on the right, we focused our search and analyses on some major types of model organisms whose genomes have been already sequenced and four comprehensive databases (GenBank, UniProt, plantTFDB and Phytozome) according to Wen method [33]. The horizontal axe does not make any sense.

MYB proteins were extensively found in plants, some fungi, bacteria, and animals [7]. Here, we focused our search and analyses on some major types of model organisms whose genomes have already been sequenced, including red algae, chlorophytes, moss, lycophyte, eudicots and monocots [30]–[32] and on four comprehensive databases (GenBank, UniProt, plantTFDB, and Phytozome) according to a previous method [33]. We identified a large number of putative *MYB* genes in angiosperms, but only a small number in earlier diverged land plants (Fig. 4). The results showed that, as a gene super family that plays important roles in regulation of developmental and defense responses pathways, *MYB* genes conservatively exist in the plant kingdom. In general, only a few *MYB* homologous genes could be found in unicellular green algae and red algae, but plants possess a large number of *MYB* genes (Fig. 4). The results indicate that the earliest evolutionary origin of the gene containing the *MYB* was from unicellular green algae of chlorophyta, suggesting that MYB proteins arose before plants transitioned from water to land. Meanwhile, this suggested that a huge expansion occurred after the evolution of land plants. With the evolution of species, the land plants have developed a series of highly sophisticated mechanisms that help them to adapt to changing environmental conditions [34]; hence, the number of *MYB* genes increased and they were extensively found in land plants in response to the environmental stimuli and regulation of physiological reactions [30].

### Chromosomal distribution, evolutionary origin and divergence of *CsMYBs*

**Figure 5:**
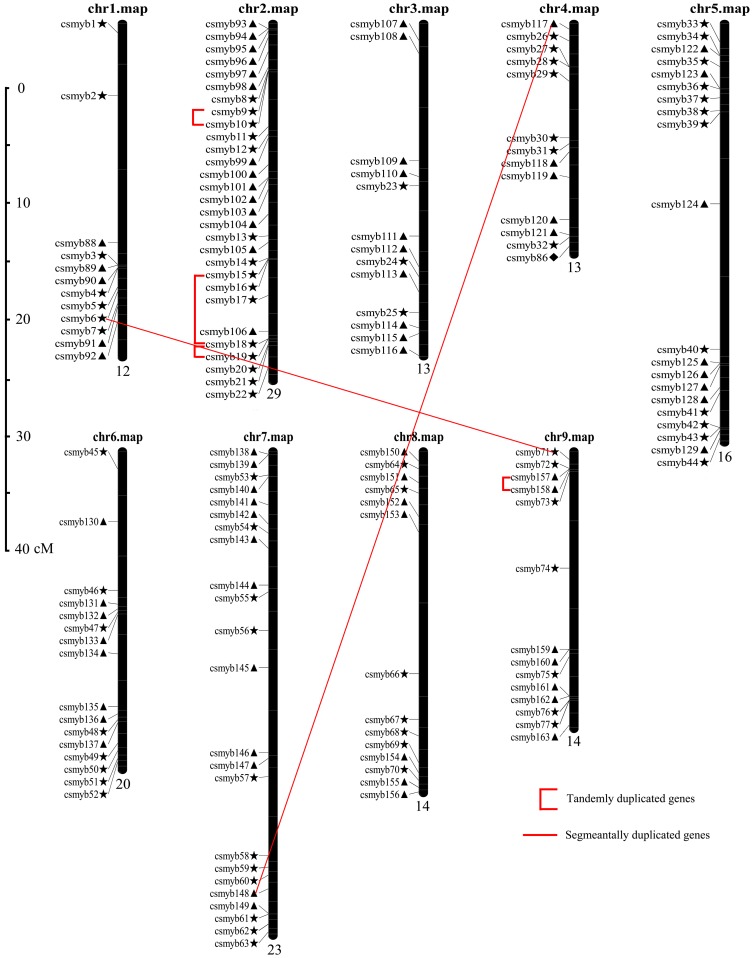
Chromosomal locations and regional duplication for *CsMYBs*. The chromosomal position of each *CsMYB* was mapped according to the sweet orange genome. The chromosome number is indicated at the top of each chromosome. The number below indicates the number of *CsMYBs* in each chromosome. The scale is 5 Mb. The bars with numbers on the chromosomes indicate the four predicted duplication regions. ▴: 1R-MYB genes; ★: R2R3-MYB genes; ⧫: 3R-MYB genes; •: 4R-MYB genes.

The position of all 177 *CsMYB*s was mapped on sweet orange chromosome available from the Citrus sinensis Annotation Project (CAP) (Fig. 5). The distribution and density of the *CsMYBs* were not uniform on all the nine chromosomes of sweet orange. Some chromosomes and chromosomal regions have a higher density of *CsMYBs* than other regions. The largest number of *CsMYBs* was located on chromosome 2 (29 *CsMYBs*), followed by chromosome 7 (23 CsMYBs), whereas chromosome 1 had only 12 *CsMYB*s. The same number of genes was located on chromosomes 3/4 (13 *CsMYBs*) and 8/9 (14 *CsMYB*); chromosomes 5 and 6 contained 16 and 20 *CsMYBs*, respectively (Fig. 5). In addition, 23 *CsMYBs* were not exactly located on the chromosome because of an incomplete physical map for sweet orange (Table S1). It should be noted that *CsMYBs* appeared on each chromosome in sweet orange. This phenomenon indicated that the distribution of *CsMYBs* was wide, which may represent the distributed characteristics of the *MYB* gene family in flowering plants.

**Figure 6:**
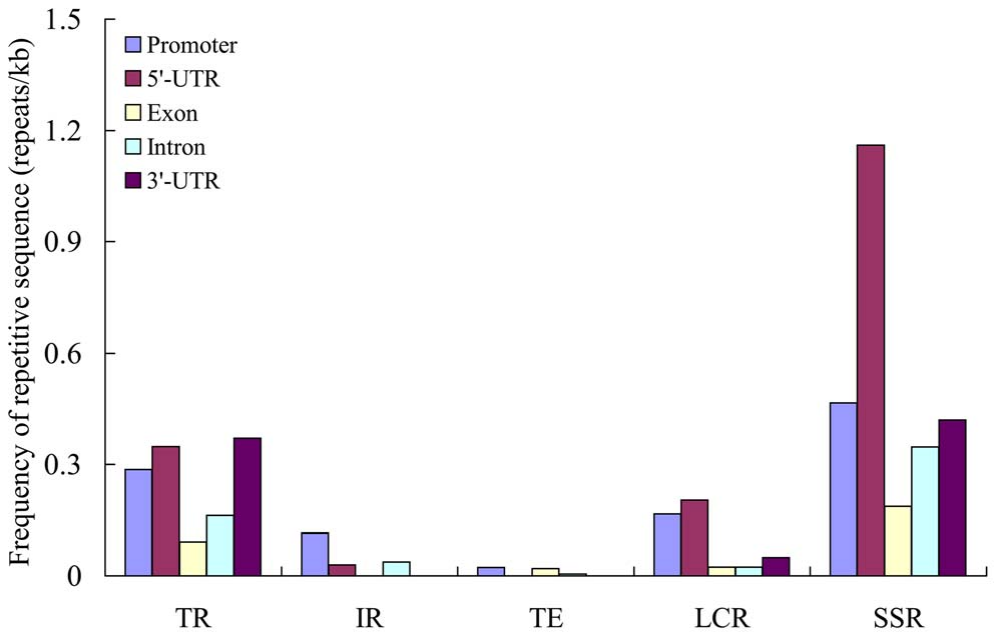
Frequency distribution of five repetitive sequences in the genomic fraction of *CsMYB*s. The number of repetitive sequences was counted for every 1 kb of promoter, 5′-UTR, exon, intron, and 3′-UTR of *CsMYBs*. TR: tandem repeat; IR: inverted repeat; TE: transposable element; LCR: low complexity repeat; SSR: simple sequence repeats.

Based on the phylogenetic relationship and sequence similarity, we identified six pairs of duplication genes with high levels of protein sequence similarity based on the following criteria: the length of aligned sequence covers>80% of the longer gene and the similarity of the aligned regions is>70% [35]. Approximately 6.21% of the entire family comprised duplication genes with sequence similarity from 72.22% (*CsMYB15* and *CsMYB18*) to 97.22% (*CsMYB18* and *CsMYB19*). Among *CsMYBs* with a high degree of homology, four (66.7%) pairs of *CsMYBs* within chromosomal segments have clear relatives in the sweet orange genome, suggesting that they may have evolved from tandem duplication events (Fig. 6). Three of those multiple pairs of duplicated regions were located at chromosome 2, and the others were distributed on chromosome 9. In particular, the putative duplications between *CsMYB9/10* and *CsMYB18/19* were highly similar. Moreover, on the putative tandem duplications of chromosome 2, the order of *CsMYB9/10* arrangement on the top arm of chromosome 2 was similar to *CsMYB18/19* at the bottom arm of this chromosome (Fig. 5).

Duplicated genes often evolved to partition existing functions or to obtain new functions, enhancing adaptability of plants [36], [37]. It is likely that different *MYB* genes evolved after duplication of their DNA-binding domain, subsequently because of a series of synonymous and/or non-synonymous mutations in the ORF, to generate new functions during evolution. Using a divergence rate of 6.5610^−9^ mutations per synonymous site per year [38], we calculated the divergence time of 67 closely related gene pairs as shown by the phylogenetic tree (Fig. 3). Table S5 showed that the divergence periods for most gene pairs were approximately 100 to 400 million years ago (MYA), with a standard deviation of 800 MYA, indicating that most gene duplication events occurred before the divergence of Malvales species (85 MYA)[28]. Divergence times of two pairs of duplicated genes, *CsMYB9/10* and *CsMYB18/19*, were estimated to be approximately 3.50 MYA and 2.43 MYA, and may represent two newly duplicated gene pairs (Table S5). On the contrary, the divergence times of three pairs of genes (*CsMYB27/79*, *CsMYB11/84*, and *CsMYB7/26*) once surpassed the divergence time of Malvales species (85 MYA; Table S5)[28]; hence, it is difficult to infer their actual divergence time. To study the selection pressures among duplicated *CsMYBs*, the substitution ratio of non-synonymous (Ka) to synonymous (Ks) mutations (Ka/Ks) was calculated for the 67 gene pairs. Ka/Ks values of most gene pairs were <1 except for two gene pairs (*CsMYB127/137* and *CsMYB173/174*), suggesting that these *CsMYB* pairs evolved under purifying selection in citrus. For *CsMYB127/137* and *CsMYB173/174*, the Ka/Ks ratio>1 indicates positive selection. Positive selection is vital in the functional divergence of protein-coding genes [39].

### Specific repetitive elements in *CsMYBs*

To characterize the sequence divergence of *CsMYBs*, the spatial distribution of repetitive sequences with respect to the genomic position of *CsMYBs* was examined. We investigated the distribution of five types of repetitive sequences frequently found in the promoter and coding regions of *CsMYBs* (tandem repeat [TR], inverted repeat [IR], transposable element [TE], low-complexity repeat [LCR], and simple sequence repeat [SSR]). To correlate repetitive sequences with specific genomic fractions (Fig. 6), we assigned five categories of sequences: promoter; 5′-UTR; coding exon; intron; and 3′-UTR. The results indicated that all the *CsMYBs* had repetitive sequence insertions and the repetitive sequences were frequent in the promoter and 5′-UTR regions (1.10 and 1.74 repeats/kb, respectively) compared with the exons (0.32 repeats/kb), introns (0.57 repeats/kb), and 3′-UTR regions (0.84 repeats/kb), primarily because of a higher frequency of SSR (Fig. 6). In addition, repeat frequency varied between the different transcribed fractions. Most of the repetitive sequences in the transcribed regions were detected in 5′-UTRs, introns, and 3′-UTRs, with the highest repeat frequency in the 5′-UTRs, which were characterized by elevated levels of TR and SSR. The frequent occurrence of repetitive sequence insertion in the promoter and transcribed regions of the genes may lead to the divergence of expression patterns and function or gene silencing to the pseudogene. Among these 337 SSRs, 166 (49.2%) were identified in promoter regions, and 75 (22.3%), 40 (11.9%), 39 (11.6%), and 17 (5.0%) SSRs were identified in intron regions, 5′-UTR regions, exon regions, and 3′-UTR regions, respectively (Table S6). Furthermore, the most abundant repeat type was bi-nucleotide repeats (146, 43.32%), followed by tri-nucleotide repeats (68; 20.18%) and tetra-nucleotide repeats (40; 11.87%), respectively (Table S7). The AT/TA motif hadthe highest frequency (66; 19.58%) followed by the motif TC/CT (31; 9.20%), AG/GA (20; 5.93%), TAA/AAT (15; 5.93%), AC/CA (15; 5.93%), and GT/GT (15; 5.93%).

To investigate whether the potential SSR loci mined were true to type and could be used for genetic analysis, 20 SSR primer pairs (Table S7) were randomly selected and verified in 11 citrus and relating accessions (clementine mandarin, sweet orange, trifoliate orange, precocious trifoliate orange, Satsuma Mandarin, kumquat, citron, iemon, grapefruit, Honghe papeda and Ichang papeda) from three genus of the Rutaceae family. The results indicated that two pairs of primers (*CsMYB96* and *CsMYB97*) could not amplify any fragment in sweet orange because of genetic variations in these primer sequences. Interestingly, all of the primer pairs (including *CsMYB96* and *CsMYB97*) generated clear DNA bands with the expected size in other citrus species and all amplicons were verified for each primer by sequencing (Fig. S4). These results suggested that the SSR markers derived from sweet orange produced a high rate of transferability among the citrus species. The transferability differed among primers; the *CsMYB37-1*, *CsMYB96*, and *CsMYB38* had lower transferability than others. In addition, the *CsMYB165* and *CsMYB59* had lower polymorphisms than others (Fig. S4). Thus, 100% of SSR primers could be used to analyze genetic diversity.

### Expression patterns analysis of *CsMYBs* in sweet orange

**Figure 7:**
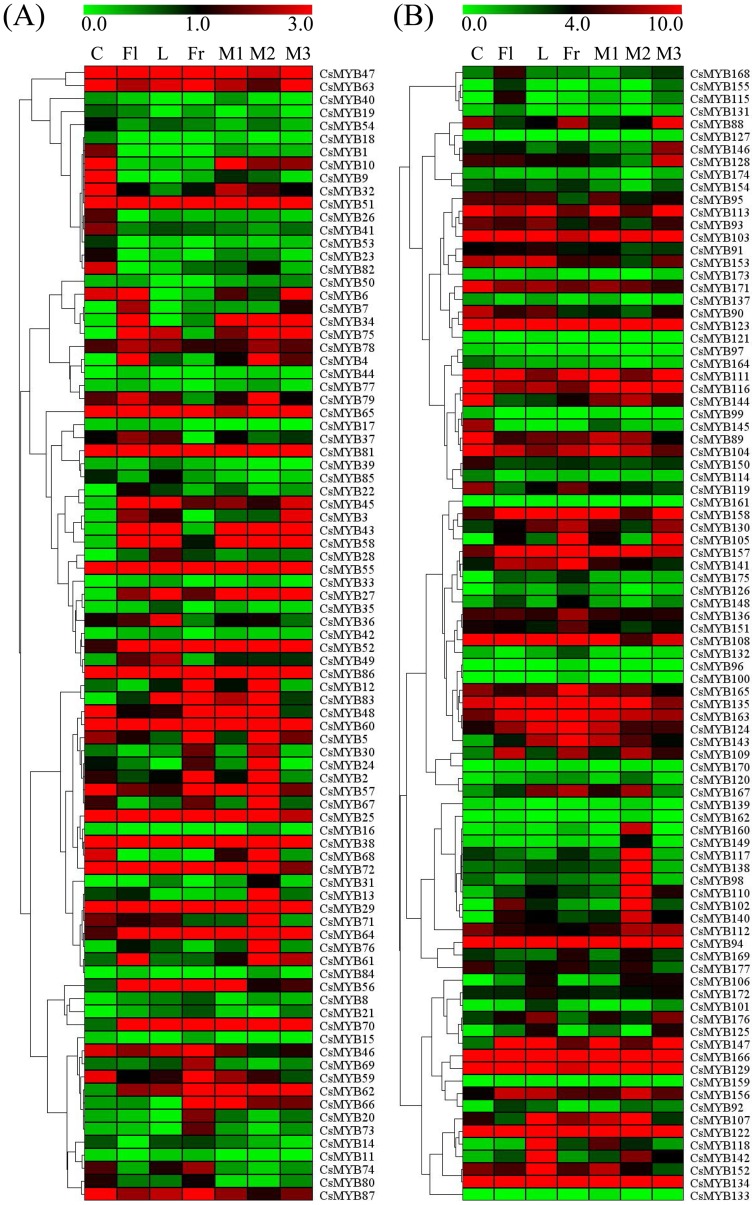
Heatmap showed the expression of *CsMYBs* in different sweet orange tissues. (A) RNA-Seq analysis of the 85 2R-MYB gene expressions, one 3R-MYB gene expression, and one 4R-MYB gene expression.; (B) RNA-Seq analysis of the 90 1R-MYB gene expressions. C: callus; Fl: flower; L: leaf; Fr: fruit; M1: mixture 1; M2: mixture 2; M3: mixture 3 (mixture indicates mixed fruit tissues from different developmental stages). Genes highly or weakly expressed in the tissues are colored red and green, respectively. The heat map was generated using cluster 3.0 software. The color scale shown at the top represents RPKM-normalized log2-transformed counts [78].

The expression patterns of genes can provide important clues for the gene function. Therefore, RNA-Seq data were downloaded CAP [40] and applied to analyze the expression patterns of *CsMYBs* in different tissues (Fig. 7). The heat map generated for examining the tissue-specific expression showed a differential transcript abundance of 177 *CsMYBs* in seven major tissues, namely callus, flower, leaf, fruit, mixture 1, mixture 2, and mixture 3 (mixture indicated mixed fruit tissues from different developmental stages). Many of the *CsMYBs* exhibited low transcript abundance level (Fig. 7); the 2R-MYB genes showed relatively higher expression compared with the 1R-MYB genes. In 1R-MYB genes, approximately 33 genes showed higher expression in all seven tissues and, conversely, 26 genes were found to have low expression (Fig. 7A). In 2R-MYB genes, approximately 31 genes showed higher expression in all seven tissues and, conversely, 36 genes were found to have low expression (Fig. 7B). Some of the *CsMYB* also showed tissue-specific expression, such as *CsMYB1/9/26/41/23/82/145*, which were expressed only in callus (Fig. 7). The tissue-specific expression profiling of *CsMYBs* would facilitate the combinatorial usage of *CsMYBs* in transcriptional regulation of different tissues, whereas ubiquitously expressed *CsMYBs* might regulate the transcription of a broad set of genes (Fig. 7). Expression pattern shifts of the duplicated paralogous genes can reflect the maintenance of duplicate genes through non-fictionalization, neofunctionalization and subfunctionalization [41]. Duplicated *CsMYBs*, two pairs of segmentally duplicated genes (*CsMYB6/71* and *CsMYB117/148*) and one pair of tandemly duplicated genes (*CsMYB9/10*), showed divergent expression profiles for duplicate genes. The *CsMYB9* and *CsMYB18* showed low expression levels in mixed fruit tissues, whereas its paralog, *CsMYB10*, was preferentially expressed in these tissues. These results indicate that the fate of the two *CsMYB* pairs could be described as neofunctionalization becausethe expression of one copy of the paralog had an obvious increase in fruit.

### Expression patterns of *CsMYBs* under stress conditions

**Figure 8:**
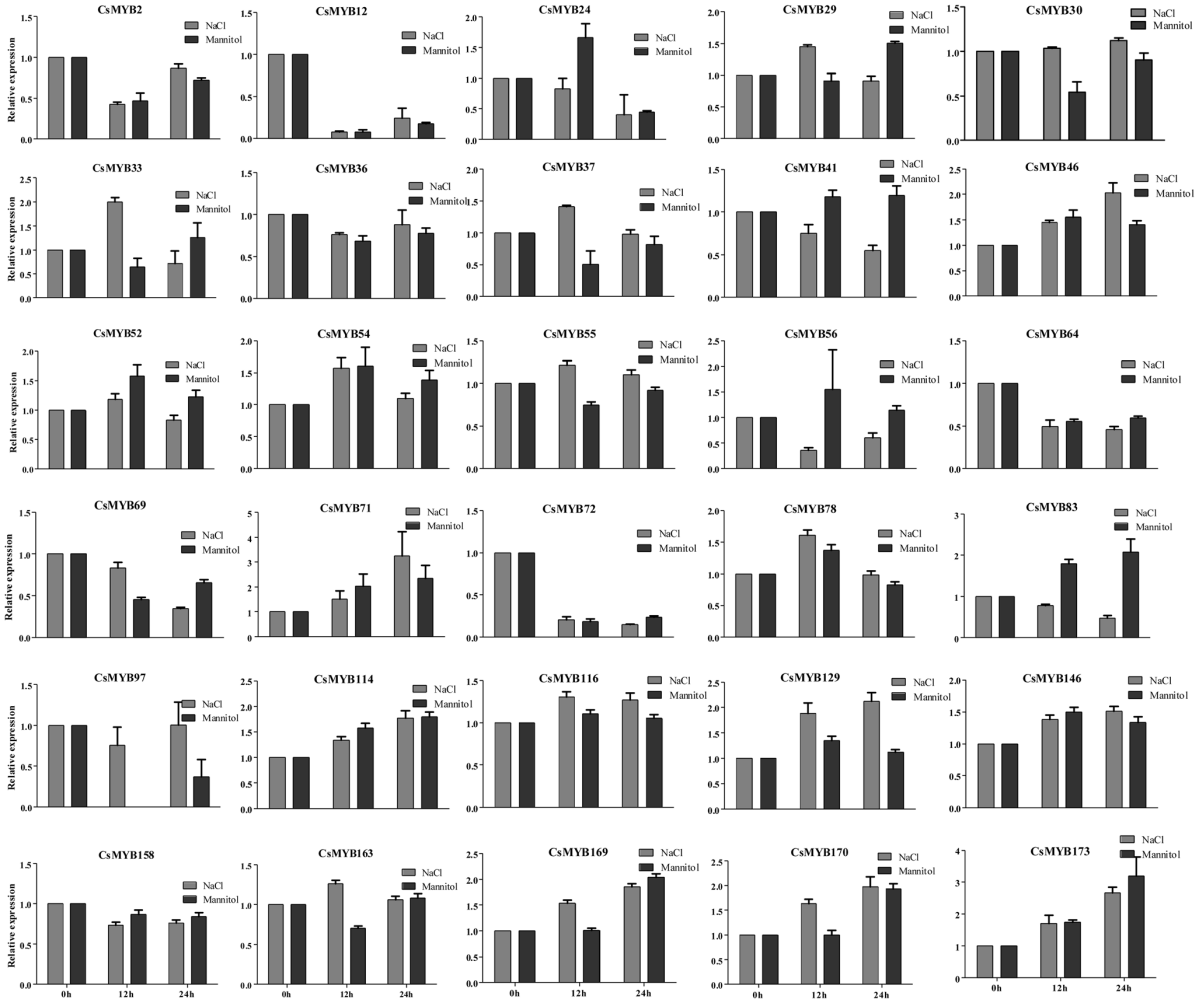
Real-time polymerase chain reaction (PCR) of *CsMYB* gene expression under NaCl and mannitol . Total RNA was extracted from sweet orange callus at 0 hours, 12 hours, and 24 hours after exposure to NaCl and mannitol, respectively. Relative transcript levels are calculated by real-time PCR with *Actin* as a standard. Data are means ± standard error (SE) of three separate measurements.

To identify *CsMYBs* with a potential role in abiotic stress response of sweet orange, the sweet orange callus was treated by NaCl and mannitol individually (Fig. 8). The expression pattern of 30 candidate genes was investigated using two treatments at 0 hours, 12 hours, and 24 hours by real-time polymerase chain reaction (PCR). These genes were selected based on phylogenetic analysis, and one gene from each cluster was selected for expression analysis. In summary, real-time PCR analyses showed that most selected *CsMYBs* have incurred variations in their expression patterns in response to one or two stresses during the course of the experiments (Fig. 8). For example, *CsMYB71/114/173* was induced by both NaCl and mannitol, whereas *CsMYB129* was only induced by NaCl. Interestingly, some genes behaved oppositely to their expression profile when subjected to two different treatments. For instance, *CsMYB33/37/55/129* were induced by NaCl but were repressed by mannitol, and *CsMYB41/56/83* were repressed by NaCl but induced by mannitol (Fig. 8). When exposed to different treatments, the expression profiles of 17 *CsMYBs* seemed to be consistent. The real-time PCR expression profiles generated revealed different expression patterns (upregulation and downregulation) for these *CsMYBs* under specific treatments, thus providing a useful resource for future gene expression and functional analyses.

## Discussion

The *MYB* gene family has been defined as the most abundant transcription factor family in plants, with at least 183 and 198 members in rice and *Arabidopsis*, respectively [9]. However, no related information has been reported regarding the whole MYB gene family in citrus. In this study, a total of 177 *CsMYBs* were isolated using the genome of sweet orange by genome-wide analysis [28]. Recently, two other full-length annotated citrus genome assemblies (sweet orange and Clementine mandarin) have been produced and have been made available to the global research community [26]; a total of 173 *MYB* genes were also indentified in the sweet orange genome (http://www.phytozome.net/) based on criteria of this study (Table S8). This study is about genome-wide classification and evolutionary and expression analyses of the *MYB* gene in sweet orange. Therefore, the *MYB* genes from the two sweet orange genomes were compared. Sequence analysis showed that most of the sequence showed high identities and only some genetic variations (including single nucleotide polymorphisms and insertion/deletion polymorphisms) in these transcripts (Table S8). However, obviously, there were more identified *MYB* genes in the former comparison than in the latter because of an incomplete genome for the latter. The former genome is from double haploids of sweet orange based exclusively on Sanger sequencing [28]. However, the latter genome assembly was produced with Sanger sequencing and 454 technology based on the Clementine mandarin genome [27], [42]; the results indicated that the former genome sequence may be more reliable and complete compared with the latter genome. Therefore, the *MYB* genes from the former were further analyzed in this study.

To obtain an overall picture of these *CsMYBs* and their relationships with *Arabidopsis*, phylogenetic trees combining the sweet orange and *Arabidopsis* MYB proteins were constructed, which divided the 177 *CsMYBs* into 42 clades. Most of the clades (38 of 42) contained different numbers of *Arabidopsis* MYB proteins, indicating the large extent of conservation among the sweet orange and *Arabidopsis MYB* gene families. So far, five *Arabidopsis* 3R-MYB genes and two 4R-MYB genes have been identified, and one of the two 3R-MYB genes positively regulates cytokinesis [43]. In this study, only one 3R-MYB gene and one 4R-MYB gene were grouped together with all five *Arabidopsis* 3R-MYB members and two 4R-MYB members. It seems that the 3R-MYB and 4R-MYB subfamilies have sequence conservation between sweet orange and *Arabidopsis*, which may be related to their conserved functions in different species. On the other hand, these results also indicated that there may be functional redundancy for 3R-MYB and 4R-MYB type proteins in *Arabidopsis*. Moreover, sweet orange and *Arabidopsis* MYB proteins were not equally represented within given clades. Six clades did not include any *Arabidopsis* MYB proteins, but they did include members only from sweet orange in this study. These results suggested the existence of species-specific *MYB* genes that were either lost in *Arabidopsis* or acquired in the sweet orange lineages after divergence from the most recent common ancestor. The anatomical and physiological differences between sweet orange and *Arabidopsis* indicated that some clades may have been differentially expanded when comparing *MYB* genes in woody and model plants. Within and outside some of these functional clades, it was common to find that two or more *Arabidopsis MYB* genes were putative orthologs of a single gene or no gene in sweet orange. This might reflect the loss of a clade member or misannotation of the sweet orange genome.

Considering the size of the sweet orange genome, the *MYB* family members of sweet orange should be approximately three-times larger than the many members of *Arabidopsis*. However, we performed comprehensive searches for putative *MYB* genes and only found 177 *CsMYBs* in sweet orange. It has long been hypothesized that understanding and distinguishing ohnologs gone missing from orthologs were pervasive problems in genomics because of multiple genome duplication events [44]. However, a previous study has indicated that sweet orange has not experienced recent whole-genome duplication (WGD) events [28]. These results indicated that reduction of the *CsMYBs* cannot be explained by WGD. Even though it is uncertain how the redundant genes were deleted, it is possible that the insertion of repetitive sequences might lead to the pseudogenization of paralogs. Analysis of repetitive sequences in the promoter and the genic regions of *CsMYBs* revealed that all *CsMYBs* contain repetitive sequence insertions. Therefore, repetitive sequence insertion possibly changes the structure of the original gene. For example, a previous study has demonstrated that transposon activation can induce the alteration of *cis*-regulatory elements or sequence loss [45]. On the other hand, LCR and SSR in duplicate genes may also become active and increase the mutation rate when the species suffers under harsh environments [46]. TR and IR sequences together promote the variation and divergence of the introns of paralogs [47]. These findings were consistent with recent reports indicating that the splicing pattern and *cis*-regulatory region of the duplicates genes changed rapidly after duplication and led to changes in the constitution of the protein [28], [48], [49]. Thus, the variation in the promoter and genic regions may possibly promote sequence divergence and gene inactivation, and may eventually result in paralog reduction in the sweet orange *MYB* gene family.

Gene duplication events contributed significantly to the proliferation of *MYB* genes in the plant kingdom [5], [14]. To further investigate gene duplication in sweet orange, tandem duplications and segmental duplications were identified. Of the 177 *CsMYB* genes, we noted that eight genes involved in tandem duplication events and four genes involved in segmental duplication events were observed. We also observed that segmental duplication and tandem duplication events were the contributors to the expansion of the *MYB* gene family in sweet orange after their divergence. These results further support gene duplication in sweet orange and *Arabidopsis* during evolution which may allow functional diversification by adaptive protein structures [50]. It is believed that multiple members of a specific gene family that form a large regulative network to control complicated physiological processes were a result of the long evolutionary history of a particular species. The individual members of a gene family represent a succession of genomic rearrangements and expansions during the process of evolution. In this study, we found at least four putative segmental duplication events in the sweet orange genome; those duplications influenced the distribution of *MYB* genes in sweet orange. In addition, we noted that four pairs of segmentally duplicated and tandemly duplicated genes showed divergent expression profiles (Fig. 7). These findings further indicate that the recently emerging genes were silenced or gained different expression characteristics compared with other members, suggesting that the birth and death of duplicated paralogs were an ongoing processes. Therefore, internal sequence divergence and natural selection can serve as ultimate factors that influence the fate of duplicated genes [51].

Currently, the most extensive annotative evaluation of plant *MYB* genes was obtained from studies of *Arabidopsis*. To evaluate the function in the sweet orange *MYB* gene family, we performed a combined phylogenetic analysis of *Arabidopsis* and sweet orange. Several CsMYB proteins were clustered into *Arabidopsis* functional clades, which provide valuable information for studying the functions of *CsMYBs*. For example, *CsMYB68*/*80* were clustered into clade 15 and shared a high level of sequence similarity with three *Arabidopsis* meristem-formation regulation proteins *AtMYB37/RAX1* and *AtMYBYB38/BIT1/RAX2* [52], [53], implying that the possible functions of *CsMYB68/80* were related to the development process of the meristem. The *CsMYB13*, *CsMYB20*, and *CsMYB82* were assembled together with *AtMYB33/65*, *AtMYB21/24*, and *AtMYB108* [54], [55] in clade 9 and clade 11, which represent the functional clades of anther regulation or stamen development. The *CsMYB39/52/53* were grouped into clade 18 with two *Arabidopsis* proteins, *AtMYB16/106* [56], [57], representing the functional clade with proteins responsible for cell development or morphogenesis. When plants are exposed to unfavorable conditions, a number of genes play roles in the response and tolerance to stresses. Clade 4, clade 17, clade 20, and clade 23 contained many of the *Arabidopsis* proteins that are involved in these processes. For example, *AtMYB30* was an activator of the hypersensitive cell death programmed in response to pathogen attack [21]. *AtMYB96* mediated the ABA signal network that confers abiotic and/or biotic stress tolerance [58]. Fourteen sweet orange 2R-MYB proteins were grouped into these clades, thus providing significant guidance to identify these genes that may putatively play roles in the response or tolerance to stress conditions. Although the 1R-MYB subfamily seemingly expanded and evolved more rapidly than the 2R-MYB subfamily in sweet orange, the phylogenetic analysis of the *Arabidopsis* 1R-MYB proteins also identified some functional clades [4], [14]. In this study, several CsMYB proteins were clustered into those clades. Because the functions of most CsMYB protein have yet to be characterized in sweet orange, it will be useful to reveal the orthologs within each clade. These results undoubtedly provide important clues for studying the function of these *CsMYBs*.

It has been noted previously that many *MYB* gene families exhibit great disparities in abundance among different organisms and different tissues to exert different physiological functions [18], [56]. Thus, gene expression patterns can provide important clues for gene function. To further analyze the tissue specificity of *CsMYBs*, we confirmed their transcription levels in seven different tissues (Fig. 7). The expression pattern of these genes suggested that most clustered gene pairs showed the same expression pattern. On the other hand, other clustered pairs exhibited different expression patterns. These results indicated that most of clustered gene pairs had more similarities in the MYB domain and shared similar expression patterns; they might be functionally redundant. Many studies have reported that the *MYB* genes play important roles in the regulation of gene expression to cope with environmental changes in plants [59], [60]. A number of *MYB* genes were characterized to function as key factors in the signaling pathways for plant resistance to abiotic stresses in the model plants [3], [61]. In this study, the expression patterns of 30 *CsMYBs* were investigated under different abiotic stress conditions. Most selected *CsMYB* genes responded to two different stress conditions in this study, indicating that they were major factors involving cross-talk among different signal transduction pathways in response to abiotic stresses. Additionally, some genes exhibited opposite expression patterns under different stress conditions. However, the gene expression profiles that respond to different stress conditions usually tend to be different, implying that the signaling pathways in the plant abiotic stress response are very complicated systems. From an applied perspective, the identification of *CsMYBs* with potential value in stress resistance improvement of sweet orange would likely benefit from targeting such genes that are part of abiotic and biotic stress response networks.

## Materials and Methods

### Plant materials

For SSR analysis of *CsMYBs*, DNA was isolated from leaves. The samples of the 11 citrus and relating accessions (Satsuma mandarin, *Citrus unshiu*; grapefruit, *Citrus paradisi*; sweet orange, *Citrus sinensis*; clementine mandarin, *Citrus clementina*; lemon, *Citrus limon*; Ichang papeda, *Citrus ichangensis*; kumquat, *Fortunella hindsii* var. chintou Swing; citron, *Citrus medica*; Honghe papeda, *Citrus honghensis*; trifoliate orange, *Poncirus trifoliata*; precocious trifoliate orange, *Poncirus trifoliata*) were collected in the experiment fields of the National Citrus Breeding Center at Huazhong Agricultural University (30°28′ N, 114°21′ E, 30 m). For NaCl and mannitol treatment experiments, in vitro callus of sweet orange was maintained at 2-week intervals on callus growth media containing Murashige-Tucker (MT) media, 3% sucrose, and 0.7% agar (pH 5.8) in the dark at 25°C. After subculture for four cycles, 2-week-old callus was used for two different experiments. Callus was cultured on callus propagation media supplemented with 100 mM NaCl and 100 mM mannitol, respectively. The callus was sampled at 0 hours, 12 hours, and 24 hours. Each experiment was repeated three times. All the samples were stored at −80°C. Total RNA and DNA were isolated as described by Zhang et al [62] and Cheng et al [63].

### Identification and gene structure of *CsMYBs*

Sweet orange genome sequences were downloaded from the Orange Genome Annotation Project (http://citrus.hzau.edu.cn/orange/index.php). To identify the maximum number of MYB domain-containing sequences, two different HMM profiles were adopted to search for *CsMYBs*. One was obtained from the Pfam database, and the other profile was generated by alignments of 198 *Arabidopsis MYB* genes [64]. Second, the Pfam database was used to determine if each of the candidate *MYB* sequences was a member of the *MYB* family. To exclude the overlapping genes, all of the candidate *MYB* genes were aligned using ClustalW [65] and confirmed manually. All of the non-overlapping *MYB* genes were used for further analysis. Information about coding sequence (CDS), full-length sequence and amino acid sequence was also obtained for each gene from the Orange Genome Annotation Project using the BLAST program [40].

The gene structure display server (GSDS) program [66] was used to draw *CsMYB* gene structures according to full-length genomes and CDS sequences from the Orange Genome Annotation Project.

### Analysis of *CsMYB* protein properties and conserved motifs

More information about nature of the CsMYB protein, GRAVY, PI, and the molecular weight was predicted by the ProtParam tool available on the Expert Protein Analysis System (ExPASy) proteomics server (www.expasy.ch/tools/protparam.html). The subcellular localization of MYB proteins was predicted by the Protein Localization Server (PLOC) (http://www.genome.jp/SIT/plocdir/)

To examine the structural divergence among the CsMYB proteins, the conserved motifs of CsMYB proteins were investigated. Their complete amino acid sequences were subjected to MEME analysis [67]. There were limitations with the software. For more detailed analyses of individual conserved motifs, the 1R, 2R, 3R, and 4R groups were analyzed separately using the MEME tool with the following parameters: optimum motif width was set from 6 to 200; and the maximum number of motifs was set to identify 15 motifs.

### Phylogenetic analysis and functional assignments of the *CsMYBs*

To examine the phylogenetic relationship and the evolutionary history of this gene family between sweet orange and *Arabidopsis*, the neighbor-joining (NJ) phylogenetic tree using bootstrap analysis (1,000 replicates) was built from the alignments of 177 CsMYB proteins and 198 *Arabidopsis* proteins. The phylogenetic trees were generated using MEGA version 4.0 [68].

The Blast2Go program [29] was run locally to BLAST against a reference database that stores UniProt entries, their associated Gene Ontology (GO), Enzyme Commission (EC), and Kyoto Encyclopedia of Genes and Genomes (KEGG) annotation [29]; the GO categorization results were expressed as three independent hierarchies for biological process, cellular component, and molecular function.

### Chromosomal locations and calculation of Ka/Ks values of *CsMYBs*

To determine the physical locations of *CsMYBs*, the starting positions of all *CsMYBs* on each chromosome were confirmed by BlastN searching using a local database of the complete sequence of the sweet orange genomes. MapChart software was used to draw the location images of *CsMYBs* [69]. To calculate the Ks and Ka values, the protein sequences were aligned and the resulting alignment was used as a reference to align the nucleotide sequences. After the removal of gaps, the Ks and Ka values were determined using the maximum likelihood method implemented in the CODEML [70] module of the PAMLpackage [71].

### Identification of specific repetitive elements of *CsMYBs*

To determine whether specific repetitive elements drive the sequence divergence of *CsMYBs*, the TR, IR, TE, LCR, and SSRs were investigated in the region 1 kb upstream of the 5′-UTR to the 3′-UTR of the genes. TRs and IRs were searched using Tandem Repeat Finder version 4.04 [72] and Inverted Repeats Finder version 3.05 [73] using default parameter values, respectively. TEs and LCRs were detected by RepeatMasker [74]. SSRs were examined with the web-based software SSRIT [75]. SSR screening and polymorphism survey were performed as described previously [76].

### Expression analysis by real-time PCR

Total RNA (3 mg) from the sweet orange was treated with 3 units of DNase (Promega, Madison, Wisconsin, USA) to remove DNA contamination and used in first-strand synthesis with an oligo (dT)_20_ primer and reverse-transcriptase according to the manufacturer's instructions (TOYOBO, Osaka, Japan). PCR primers were designed to avoid the conserved regions. Primer sequences are shown in detail in Table S7. Real-time PCR was performed by using the SYBR Green PCR Master Mix (Roche Applied Science, Mannheim, Germany) as described previously [77]. Three biologic repeats and four technical repetitions were assayed in this study, resulting in similar trends. Data from one biologic repeat are presented.

## Supporting Information

Figure S1 **Fifteen putative conserved motifs were identified in the 2R-MYB and 3R-MYB proteins using MEME search tool.** Different motifs were indicated by different colors. The same number in different groups refers to the different motif. The length of the motif in each protein represents the actual length and motif sizes are indicated in Table S3.
(DOC)

Figure S2 **Fifteen putative conserved motifs were identified in the 1R-MYB and 4R-MYB proteins using MEME search tool.** Different motifs were indicated by different colors. The same number in different groups refers to the different motif. The length of the motif in each protein represents the actual length and motif sizes are indicated in Table S4.
(DOC)

Figure S3 **Characterization of 177** ***CsMYBs*** **by gene ontology categories.** A: Biological process. B: Molecular function. C: Cellular component.
(DOC)

Figure S4 **Transferability of** ***CsMYBs*** **derived simple sequence repeats among different genomes of citrus species.** The figure between brackets represents the length of the amplified fragment. Lane 1: Satsuma mandarin; lane 2: grapefruit; lane 3: sweet orange; lane 4: clementine mandarin; lane 5: lemon; lane 6: Ichang papeda; lane 7: kumquat; lane 8: citron; lane 9: Honghe papeda; lane 10: trifoliate orange; lane 11: precocious trifoliate orange.
(DOC)

Table S1 ***CsMYBs*** **encoding MYB proteins along with their molecular details.**
(XLS)

Table S2 **MYB-domain based characterization and comparison of** ***CsMYBs*** **in terms of GRAVY, molecular weight and cellular localization.**
(XLS)

Table S3 **Analysis and distribution of conserved motifs in 1R-MYB genes.**
(XLS)

Table S4 **Analysis and distribution of conserved motifs in 2R-MYB genes.**
(XLS)

Table S5 **Estimated divergence period of** ***CsMYB*** **pairs in sweet orange.**
(XLS)

Table S6 **List of** ***CsMYB*** **-SSR markers along with their motifs and location in the gene.**
(XLS)

Table S7 **Details of primers used in the SSR and RT-PCR analysis in this study.**
(XLS)

Table S8 **Similarity analysis of sweet orange MYB gene family fron two different databases.**
(XLS)
