# Supplementary material for: Genome-Wide Classification and Evolutionary and Expression Analyses of Citrus MYB Transcription Factor Families in Sweet Orange
Source: PLoS One. 2014 Nov 6;9(11):e112375. doi: 10.1371/journal.pone.0112375 (PMC4223058; doi:10.1371/journal.pone.0112375)
Supplement: Figure S1 — Fifteen putative conserved motifs were identified in the 2R-MYB and 3R-MYB proteins using MEME search tool. Different motifs were indicated by different colors. The same number in different groups refers to the different motif. The length of the motif in each protein represents the actual length and motif sizes are indicated in Table S3. (DOC) [file pone.0112375.s001.doc]

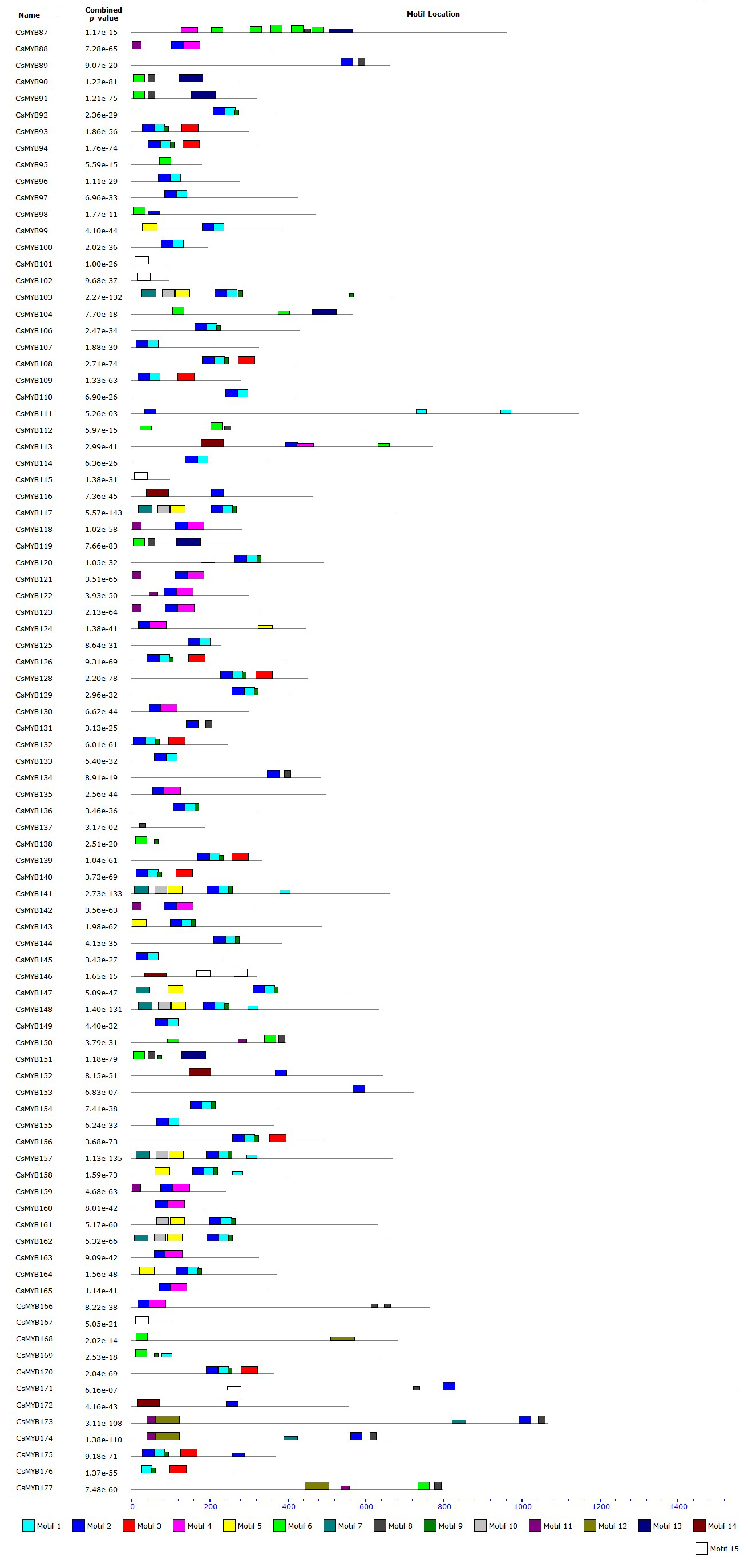


**Figure S1**: Fifteen putative conserved motifs were identified in the 2R-MYB and 3R-MYB proteins using MEME search tool. Different motifs were indicated by different colors. The same number in different groups refers to the different motif. The length of motif in each protein represents the actual length and motif sizes were indicated in table S3.
