# Supplementary material for: Genome-Wide Classification and Evolutionary and Expression Analyses of Citrus MYB Transcription Factor Families in Sweet Orange
Source: PLoS One. 2014 Nov 6;9(11):e112375. doi: 10.1371/journal.pone.0112375 (PMC4223058; doi:10.1371/journal.pone.0112375)
Supplement: Figure S3 — Characterization of 177 CsMYBs by gene ontology categories. A: Biological process. B: Molecular function. C: Cellular component. (DOC) [file pone.0112375.s003.doc]

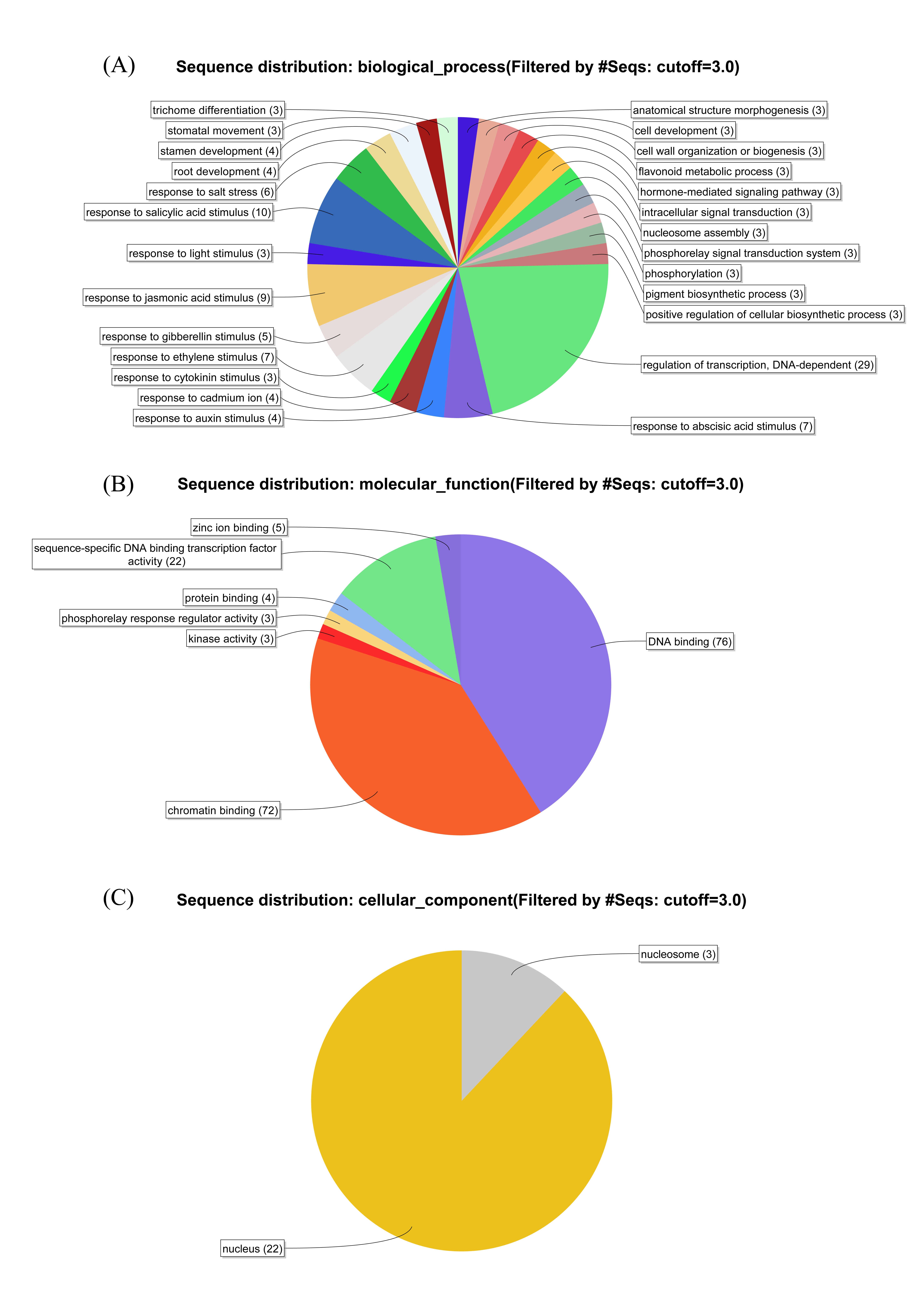


**Figure S3**: Characterization of 177 *CsMYBs* by gene ontology categories, A: Biological process; B: molecular function; C: cellular component.
