# Supplementary material for: Genome-Wide Classification and Evolutionary and Expression Analyses of Citrus MYB Transcription Factor Families in Sweet Orange
Source: PLoS One. 2014 Nov 6;9(11):e112375. doi: 10.1371/journal.pone.0112375 (PMC4223058; doi:10.1371/journal.pone.0112375)
Supplement: Figure S4 — Transferability of CsMYBs derived simple sequence repeats among different genomes of citrus species. The figure between brackets represents the length of the amplified fragment. Lane 1: Satsuma mandarin; lane 2: grapefruit; lane 3: sweet orange; lane 4: clementine mandarin; lane 5: lemon; lane 6: Ichang papeda; lane 7: kumquat; lane 8: citron; lane 9: Honghe papeda; lane 10: trifoliate orange; lane 11: precocious trifoliate orange. (DOC) [file pone.0112375.s004.doc]

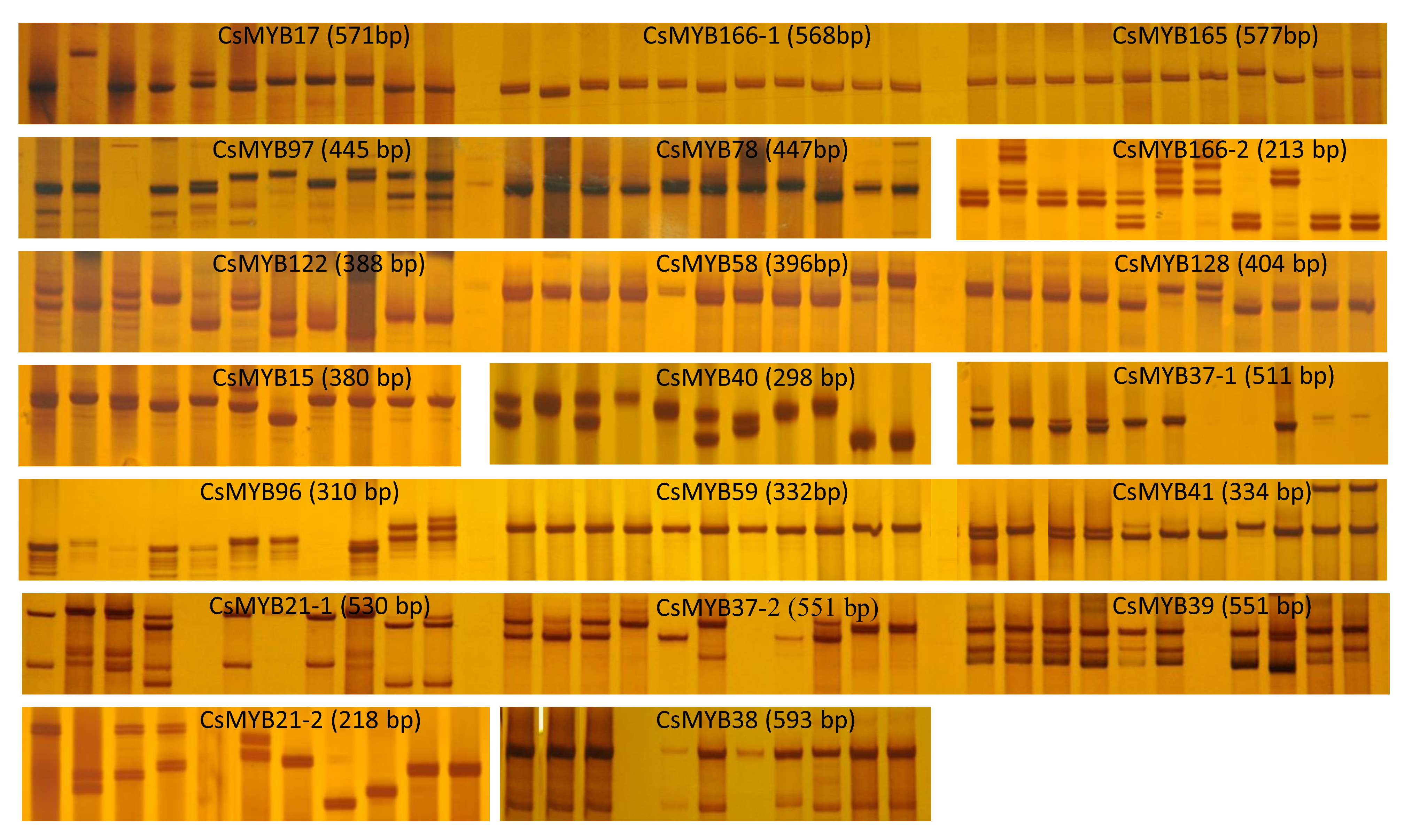


Figure S4: Transferability of *CsMYBs* derived simple sequence repeats (SSRs) among different genomes of citrus species. The figure between brackets represents the length of the amplified fragment. Lanes: 1 Satsuma mandarin, 2 Grapefruit, 3 Sweet orange, 4 Clementine mandarin, 5 Lemon, 6 Ichang papeda, 7 Kumquat, 8 Citron, 9 Honghe papeda, 10 Trifoliate orange, 11 Precocious trifoliate orange.
